# A Markov chain method for counting and modelling migraine attacks

**DOI:** 10.1038/s41598-020-60505-5

**Published:** 2020-02-27

**Authors:** Mathias Barra, Fredrik A. Dahl, Kjersti Grøtta Vetvik, E. Anne MacGregor

**Affiliations:** 1 Akershus University Hospital HF, The Health Services Research Unit – HØKH, 1478 Lørenskog, Norway; 2 Oslo University Hospital, C3 – Centre for Connected Care, 0450 Oslo, Norway; 3 Institute of clinical medicine, Campus Ahus, Univeristy of Oslo, 1478 Oslo, Norway; 4 Akershus University Hospital HF, Department of Neurology, 1478 Lørenskog, Norway; 5 Barts and the London School of Medicine and Dentistry, Centre for Neuroscience and Trauma Blizard Institute of Cell and Molecular Science, E1 2AT London, United Kingdom; 6 St. Bartholomew’s Hospital, Centre for Reproductive Medicine, EC1A 7BE London, United Kingdom

**Keywords:** Epidemiology, Migraine

## Abstract

To ensure reproducibility in research quantifying episodic migraine attacks, and identifying attack onset, a sound theoretical model of a migraine attack, paired with a uniform standard for counting them, is necessary. Many studies report on migraine frequencies—e.g. the fraction of migraine-days of the observed days—without paying attention to the number of discrete attacks. Furthermore, patients’ diaries frequently contain single, migraine-free days between migraine-days, and we argue here that such ‘migraine-locked days’ should routinely be interpreted as part of a single attack. We tested a simple Markov model of migraine attacks on headache diary data and estimated transition probabilities by mapping each day of each diary to a unique Markov state. We explored the validity of imputing migraine days on migraine-locked entries, and estimated the effect of imputation on observed migraine frequencies. Diaries from our patients demonstrated significant clustering of migraine days. The proposed Markov chain model was shown to approximate the progression of observed migraine attacks satisfactorily, and imputing on migraine-locked days was consistent with the conceptual model for the progression of migraine attacks. Hence, we provide an easy method for quantifying the number and duration of migraine attacks, enabling researchers to procure data of high inter-study validity.

## Introduction

Research into migraine disorders frequently needs to quantify aspects of episodic migraine attacks. Salient features of any migraine attack are its *onset* and its *duration*. Furthermore, headache is—to most patients—the cardinal symptom. Therefore, for the basic researcher interested in inquiring into the pathophysiology of migraine, a sound theoretical model of the entity ‘a migraine attack’ is important for grounding research, ensuring reproducibility and a uniform standard for counting, and for communicating about the occurrence of migraine attacks and their patterns.

In this paper we review key contributions from the literature on how to temporally delineate a migraine attack. The main objective is to next develop a simple, mathematical model for their study. In particular, we wish to elaborate on how the most current knowledge about the course of a migraine attack translates into a certain, well-defined, standard for recording attack data.

To this end we quantify the conditional probability of the occurrence of a migraine day continuing into the next day. Once we have an informed idea about this probability, it is possible to fix a certain, parametrised class of models known as Markov chain models, and argue for its adequacy for a specific type of migraine research. More precisely, we test the robustness of one assumption some migraine researchers rely on: that the probability of experiencing a migraine headache on any given day is independent of the previous day’s ‘migraine status’^1,2^.

This assumption has broader implications if adopted to investigate migraine triggers. For example, if a patient reports on three consecutive days migraine on the first, no migraine on the second, and migraine again on the third day, a clear standard for how to analyse these data in relation to the presence or absence of candidate triggers is clearly crucial.

### The notions of Migraine *attacks* versus migraine days

In research aimed at alleviating symptoms once an attack is already ongoing, it might be appropriate to count migraine days, since how many days a patient was inhibited by migraine is what matters. In contrast, research that aims to track how and when migraine attack onsets occur—perhaps with a view towards arresting them—and how long they last, clearly demands identifying the first day of a migraine attack. This important point bears on the difference between focusing on the underlying cause of the migraine, rather than treating the symptoms.

The work presented here was originally conceived with the aim of studying *menstrual migraine (MRM)*^3^, and our data and terminology, as well as choice of temporal unit (*a day*) reflects this progeny. Of course, much of what has been said above would apply equally to the study of other headache disorders (e.g. cluster headaches), or other intermittent conditions in general, as long as the notion of an attack with variable duration is a clinically prominent feature. In headache-research and clinical practice the *headache diary*—often a list of consecutive days on which the patient records headaches—is a commonly encountered data format, and this has informed our formalisation and nomenclature. The general ideas, however, are transferable also to other attack-natured conditions and phenomena by a suitable modification of our framework—e.g. by counting *rash*-days in psoriasis—or other headache diary formats—e.g. by counting headache-*hours* in more detailed records. We return to this point in the discussion—or to other headache diary formats.

### The notion of *Migraine-locked* days

While detailing our proposed Markov chain model, we also argue that migraine days separated by a single *migraine-locked* day—a day in a patient’s migraine diary that is immediately preceded by and succeeded by migraine days—should routinely be considered as a single attack that includes the migraine-locked day.

We emphasise that the issue of how to treat the migraine-locked days is orthogonal to the issues about how suitable our model is for reasoning about attack-natured conditions. The conceptual model we develop and recommend can accommodate the analysis of the progression of migraine attacks regardless of what position one might take on migraine-locked days. Furthermore, forming an opinion about whether the concept of migraine-locked days has applications beyond migraine research, and could be generalised to ‘*X*-locked days’ for some other condition *X* is not an objective for this work.

Our objective is to introduce a very simple, but sufficiently general, model for modelling attack-natured headache conditions. The intended application is the study of migraine and its triggers^3^. A secondary objective is therefore to describe and argue for a theoretically plausible, simple, and practice-compatible manner for counting migraine attacks. The latter objective is treated in the first two subsections of the Methods section. Subsequently, we present the case for a simple Markov chain model for migraine progression, followed by empirical estimation of the necessary number of states and transition probabilities.

## Methods

### Theory

#### Imputing migraine on *migraine-locked* days

This issue does not necessitate any new hypotheses; it merely provides an alternative way of categorising days as ‘migraine days’ that is more in accord with the pathophysiology of migraine. Furthermore, it highlights that the hypothesis that each day’s migraine probability is independent of the previous day might be too simplistic.

When identifying discrete migraine attacks based on consecutive migraine days, it is important to consider the count in context of a migraine attack. But what defines a migraine attack? Patients typically record headache days. Accordingly, the current International Classification of Headache Disorders (ICHD-3) bases the diagnosis of a migraine attack on the clinical characteristics including duration of the headache together accompanying features and the presence or absence of aura^4^. The diagnostic criteria do not account for the interruption of headache pain following effective treatment in the context of the progression of an attack. Yet it is recognised that the migraine process persists despite effective treatment for headache as shown by enduring brain stem activation during spontaneous migraine attacks following relief of headache and related autonomic symptoms with e.g. sumatriptan^5^. By convention, in the guidelines for controlled trials of drugs in migraine, the International Headache Society considers that ‘*after 2h pain freedom, any headache pain from 2 to 48h after study drug administration, regardless of its severity, should be considered a relapse*’, i.e. part of the same attack^6^. Hence, we argue that in a patient’s migraine diary, so-called *migraine locked days*—days that are immediately preceded by and immediately succeeded by migraine days—should be regarded as migraine days, thus representing fewer migraine attacks, but of longer duration (see Table 1).

**Table 1:** Migraine-locked days.

	Raw headache diary data										Counts	
Diary day:	29	30	31	32	33	34	35	36	37	$\cdots$	Days:	9
											Migraine days:	4
Migraine:		M		M			M	M		$\cdots$	MLD:	1
											Migraine attacks:	3
	Data with imputed migraine locked days										**Counts**	
Diary day:	29	30	31	32	33	34	35	36	37	$\cdots$	Days:	9
											Migraine days:	5
Migraine:		M	M	M			M	M		$\cdots$	MLD:	0
											Migraine attacks:	2

Illustration of the Fill48-, or, imputation of migraine locked days- method for the accounting of migraine days and migraine attacks. The top panel shows an excerpt of a hypothetical migraine diary: the top row records the day’s number, the bottom row records migraine headache days. The day (numbered 31) is a migraine locked day: both the day preceding it and the day succeeding it is recorded with a migraine. The lower panel shows the diary post-imputation: the migraine-locked day is recorded as a migraine day (in italics). Both panels are accompanied with a table recording the relevant counts (from the excerpt from days 29–37): in the top diary—without imputed migraine locked days—we count a total of four migraine days distributed between 3 migraine attacks. In the bottom diary—with an imputed migraine locked day—we count a total of five migraine days, distributed between 2 migraine attacks. For the researcher focused on triggers, the raw data suggests importance of days 30, 32 and 35 as migraine onsets. If the Fill48 method is employed, only days 30 and 35 counts as migraine onset days.

#### Quarantining pre-headache days

A further point to consider is the duration of migraine headache versus the duration of the migraine attack, given that the headache phase of a migraine attack is only part of the attack process. According to current understanding a migraine attack results from dysfunctional changes in the brain stem, cortex and hypothalamus which starts at a certain point in time^7^. The pathophysiological changes follow a continuous process, which last for a period of time, until the attack has subsided. From the clinical perspective a migraine attack begins with premonitory symptoms followed by headache and ending with the postdromal phase; aura, if present, precedes headache but is discrete from the more generalised premonitory symptoms^8^. So when considering the onset of an attack and its duration, it is important to consider the entire migraine process, not just the headache. While both a premonitory phase occurring hours or days before the headache, and a resolution phase following headache are noted in the ICHD-3, they are not a requirement of the diagnosis of migraine and are not accounted for in the attack duration^4^. We argue that if an attack is already in progress before patients record headache, those days should not be counted as interictal days but should be quarantined as part of the attack process.

Few studies have addressed the duration of the pre-headache brain dysfunction. A study of 76 patients (95% women) using an electronic diary to record migraine symptoms over 12 weeks found the duration of premonitory symptoms to be at least 24 hours before onset of headache and postdromal symptoms, although typically resolving within 6 hours of the headache, continued in some patients for longer than 24 hours^9^. A study comparing *contingent negative variation* (CNV) in 45 migraine patients (75% women) with 20 healthy subjects (80% women) found the amplitude of the early CNV component was more increased and its habituation was more reduced in the stress condition, especially 1–3 days before an attack compared with changes of CNV amplitudes and habituation under stress obtained after an attack, during the headache-free interval, or in healthy controls^10^. A study of blinded paired quantitative EEG in 40 migraineurs (93% women) found frontocentral *δ* power increased and a tendency towards frontocentral *α* and *θ* power increasing within 36 hours before the next migraine attack compared with the interictal period^11^. A visual luminance threshold study of 18 migraineurs (83% women) found a significant weakening of centre-surround contrast suppression starting around 48 hours prior to onset of migraine headache, which returned to headache-free levels by 24 hours post-migraine^12^. Continuous scanning of a migraine patient over thirty days during which three migraine attacks occurred found that hypothalamic activity as a response to trigeminal nociceptive stimulation was altered during the 24 h prior to pain onset^13^.

While the numbers of patients included in the studies are small, collectively the results are similar and, in the absence of more robust data, suggest that it is reasonable to assume that the 48 hours before onset of migraine headache should be quarantined.

In practice, for diaries recording migraine *days*, the number of *hours* between migraine events cannot be directly observed. Therefore, we develop a framework for analysing headache diaries in which migraine locked days are imputed as migraine days. This means that the quarantine period can be anything between 24 and 72 hours. As this period is centred on 48 hours, we call this method the *Fill48*-method throughout this work.

#### The case for Markov chains as a model for the progression of migraine attacks

**Figure 1:**
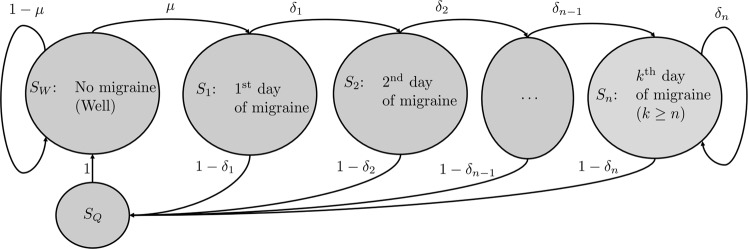
Example of an (*n* + 2)-state Markov chain representing the progression of migraine attacks. The *μ* represents the migraine onset probability of an attack during a given migraine free day. The *δ*_*i*_’s represents the probability that a migraine attack will continue into the next day, conditional on an attack already having been ongoing on the previous i days. The complementary probabilities 1 − *δ*_*i*_ are the probabilities that the migraine attack ends during that day, rather than continue into the next. The state *S*_*Q*_ represents the first full migraine free day following a migraine day—a necessary precondition for (the model) to declare that a new migraine attack can begin assuming the Fill48 assumption.

A discrete-time finite *Markov-process*, or finite *Markov chain*, is a random process characterized by the changing between finitely many *states* (e.g. representing *well* and *ill*), according to certain *transition probabilities (TPs)*^14^. Figure 1 gives a schematic illustration of a Markov chain that can represent the progression of a migraine attack.

We propose that this Markov chain will serve as a good approximation for the progression of migraine attacks. This model’s interpretation is as follows:

On any one given day, whether or not migraine attack onset occurs is—in the model—determined by the probability *μ*: the migraine onset probability. If migraine attack onset happens during the day, the Markov chain’s state is altered from *S*_*W*_ to *S*_1_. Once in this state, the probability that the migraine continues on the next day is *δ*_1_, in which case the Markov chain transitions to state *S*_2_ (interpretation: the second day of a migraine attack). Alternatively—with probability 1 − *δ*_1_—the patient’s migraine attack ceases, and the process returns to the quarantine-state *S*_*Q*_. In this Markov chain model, this migraine free day is what defines the end of the migraine attack. Once the Markov chain transitions to *S*_*Q*_, the next step is pre-determined as the transition back the initial state *S*_*W*_.

In general, if the process is currently in state *S*_*i*_—i.e. on the *i*^th^ day of a migraine attack, the migraine attack continues into the next day with probability *δ*_*i*_; equivalently, ceases and passes to *S*_*Q*_ with the complementary probability of 1 − *δ*_*i*_. (In the case that the Fill48 method is not used—but we do not recommend this—the *S*_*Q*_ transition is omitted, and the *δ*_*i*_ transitions on Fig. 1 lead directly to *S*_*W*_.)

In theory, this gives a process with infinitely many states. In practice, we can assume that after some point the empirical probability of observing one more day of headache is virtually constant (and very low), which allows for truncating the model to *n*  + *2* states for some fixed (possibly large) *n*. This truncation is reflected in Fig. 1 by an arrow looping from the migraine-state *S*_*n*_ back to itself.

At this point it is important to note that the quarantine state can simply be omitted from the model if one does not subscribe to the rationale for ignoring migraine locked days when accounting for attack duration. Rather, it should be thought of here as a case study of the models versatility. The issue of the conditional probability of seeing one more migraine day, and the need for explicitly stating ones underlying assumptions about how migraine attacks progress, is equally important whether or not this feature of the model is used. Indeed, research on migraine triggers or the prevention of migraine attacks presupposes close attention to at what times the patient at risk, undergoing an attack, or possibly non-susceptible.

### Empirical testing

In the empirical part of this study we estimate transition probabilities for the Markov chain, and explore the evidence in favour of simplifying the model. The aim of this analysis is to establish and quantify the tendency of clustering of migraine days in patients’ headache diaries, but will also provide a model for studying migraine triggers. We also explore the apparent validity of imputing migraine days on migraine-locked entries in headache diaries, and estimate the effect of data manipulation on the overall outcomes of migraine frequencies in a data set of headache diaries.

#### Data

We used a data set of headache diaries from 165 women attending the City of London Migraine Clinic during the period 1998–1999. The patients were not using any hormone treatment, continued their usual migraine treatment and were routine patients, not selected for any association between migraine and menstruation.

#### Estimation of the transition probabilities (TPs)

Each day of each diary was mapped to a unique state in the Markov chain. For example, in the hypothetical headache diary excerpt displayed in Table 1, the mapping of days to Markov chain state *S*_*W*_ were the days numbered 29, 30, and 35, since these are days when a migraine onset could occur. Since days 30 and 35, but not day 29, were recorded as migraine days, the estimated $\widehat{\mu }$ for this diary is $\widehat{\mu }=\frac{2}{3}=0.67$ (see Table 2).

**Table 2:** Transition probability estimation algorithm.

	Data with imputed *migraine locked days*										
Diary day:	$\cdots$	29	30	31	32	33	34	35	36	37	$\cdots$
Migraine:	$\cdots$		M	${\bf{M}}$	M			M	M		$\cdots$
	**Step 1: mapping of diary days to states**										
Diary day		Markov chain state			Diary day				Markov chain state		
	$\mapsto$	${S}_{W}$			34	$\mapsto$			${S}_{Q}$		
	$\mapsto$	${S}_{W}$			35	$\mapsto$			${S}_{W}$		
	$\mapsto$	${S}_{1}$			36	$\mapsto$			${S}_{1}$		
	$\mapsto$	${S}_{2}$			37	$\mapsto$			${S}_{2}$		
	$\mapsto$	${S}_{3}$									
	**Step 2: Estimate transition probabilities**										
		$\hat{\mu }$	${\hat{\delta }}_{3}$			${\hat{\delta }}_{1}$	${\hat{\delta }}_{2}$				
		$\frac{2}{3}=0.67$	$\frac{2}{2}=1.00$			$\frac{1}{2}=0.50$	$\frac{0}{1}=0.00$				

Illustration of transition estimation calculations: (1) first map each diary day to a Markov chain state; (2) then compute the frequency of the transitions.

For our data, we estimated overall TPs *μ*, *δ*_1_, …, *δ*_*n*_, where *n* was the largest number of days of an ongoing migraine attack observed in the data. This was done for both the raw data and for the set of Fill48-processed diaries. Next, $\widehat{\mu },\ {\widehat{\delta }}_{1},\ldots ,{\widehat{\delta }}_{n}$ were estimated as Bernoulli probabilities with 95% CI’s with continuity correction^15^, and by fitting a logistic regression model *M*_*j*,*d*_ ~ ***β****X*_*j*,*d*,*k*_ where the *M*_*j*,*d*_’s were coded as 1 if patient *j* recorded a migraine day on day *d* (0 otherwise), and *X*_*j*,*d*,*k*_ was coded as 1 if patient *j*’s *d*^th^ day was preceeded by axactly *k* migraine days.

The main focus of this study—the tendency of clustering of migraine days—were assessed by assessing the differences between the TPs. Furthermore, individual TPs *μ*_*j*_, *δ*_1,*j*_, …, *δ*_*n*,*j*_ for each patient *j* were estimated, and we assessed the variance of the individual TPs and the generality and face validity of the resulting Markov chain.

Note that when mapping diary-days to Markov chain-states in continuous time, at any time during a day a migraine headache is recorded that day’s state is the next migraine-state (some *S*_*i*_). However, only if no migraine was recorded during the entire day does the chain move to the next non-migraine state (*S*_*W*_ or *S*_*Q*_—we regard *S*_*W*_ as the ‘next’ non-migraine state after itself).

#### Migraine locked days and their frequencies

We counted the number of migraine locked days, computed descriptive statistics, and inspected their distribution across the patients in the data.

All statistical analyses were performed with the statistical software R (v.3.6.1, 2019-07-05) within RStudio platform; plots were generated with ggplot2 and plotly^16–19^.

### Ethics

Ethical consideration was obtained for this study from Queen Mary Ethics of Research Committee, the result of which was the conclusion that the proposed work does not present any ethical concerns; is extremely low risk; and thus does not require the scrutiny of the full Research Ethics Committee. All data analysed in this study were fully anonymised (headache diaries consisting of numbered days with records of migraine events, paired with the age and sex of the patient).

### Accession codes

The raw, anonymised headache diaries are available from https://www.researchgate.net/publication/335577175_Headache_Diaries_DATABASE.

## Results

The 165 diaries contained a total of 17 835 logged diary days, with a median of 85 days per diary, with Inter Quartile Range (IQR): 71–128. All patients were women, and mean age was 40.8 years. Pre-imputation, the raw data contained 2 303 migraine days, distributed between 1 347 migraine attacks, and with a total of 140 migraine locked days. Imputing migraine days according to Fill48 resulted in 2 343 migraine days, and the number of attacks was reduced to 1 207.

The overall frequency of migraine locked days was 0.24 per 30 observed days (see Table 3).

**Table 3:** Descriptive statistics for the migraine diaries in the data set.

	Median*	(IQR)*	Mean*
Diary-days observed	85.0	(71—128)	108.1
Migraine locked days per 30 days	0.0	(0–0.4)	0.2
Age (of the patients in years)	42.0	(35–47)	40.8
**Raw data**^†^	**Median**	**(IQR)**	**Mean**
Migraine days	11.0	(7–17)	14.0
Migraine attacks	6.0	(4–10)	8.2
Migraine days per 30 days	3.5	(2.7–4.8)	3.8
Migraine attack duration^‡^	1	(1–2)	1.7
**Fill48 data**^†^	**Median**	**(IQR)**	**Mean**
Migraine days	12.0	(7–19)	14.8
Migraine attacks	5.0	(4–9)	7.3
Migraine days per 30 days	3.6	(2.9–5.1)	4.0
Migraine attack duration^‡^	1	(1–3)	2.0

*The statistics reported (e.g. the means) are taken over the individual patient-headache diaries. ^†^*Raw data* here means that migraine locked days were not imputed into the diaries; *Fill48 data* are w.r.t. diaries with imputed migraine locked days. ^‡^I.e. the number of consecutive days of migraine—see also Table 2.

**Figure 2:**
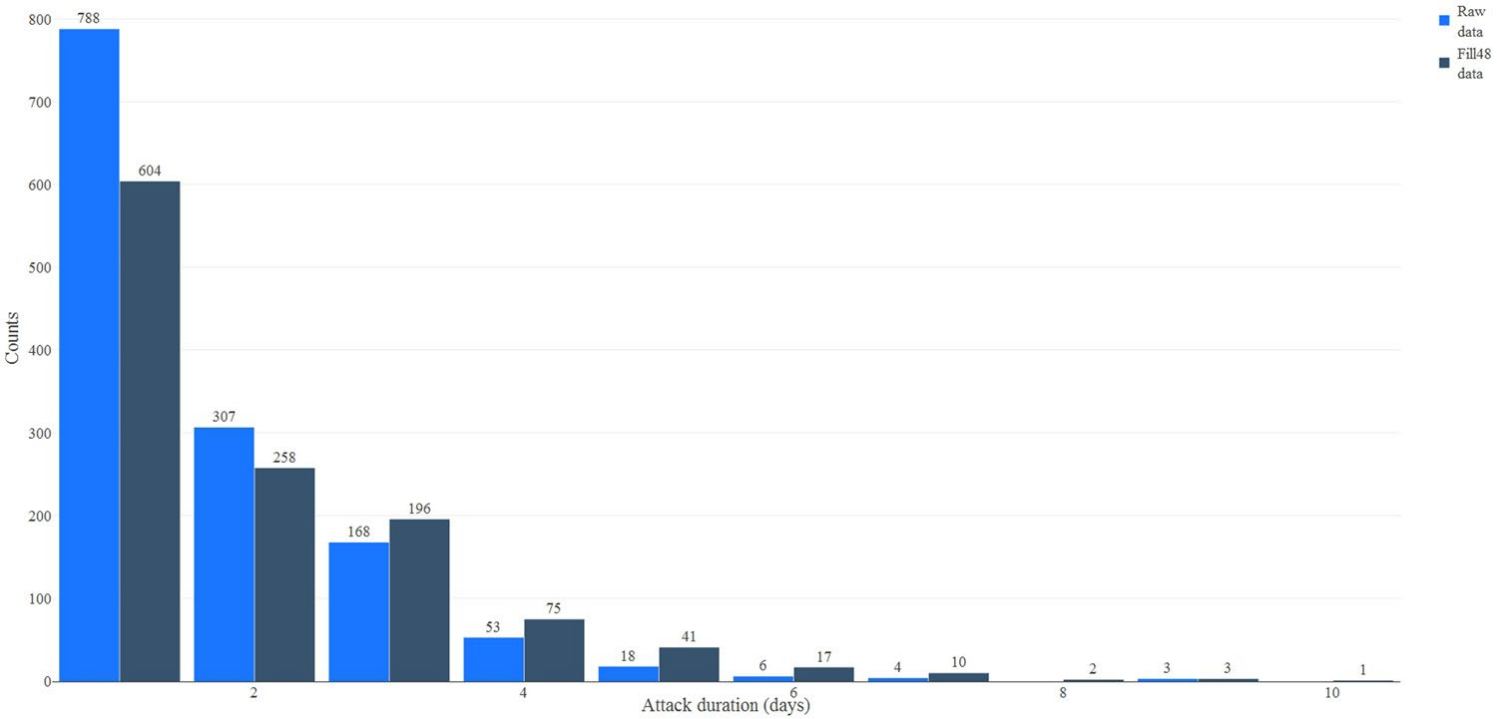
Barplot of the migraine attack durations. The *x*-axis gives the attack duration (in days); the *y*-axis give the counts. Thus, the heights of the bars represent the number of observed attacks (across the 165 headache diaries) of the durations (in days). See Table 3 for further descriptive statistics.

Most migraine attacks in the raw diaries lasted one day: 788 of the 1347 attacks (58.5%; see Fig. 2). Imputing with the Fill48 method reduced the number of one-day attacks to 604 out of the 1207 attacks (50.0%).

**Figure 3:**
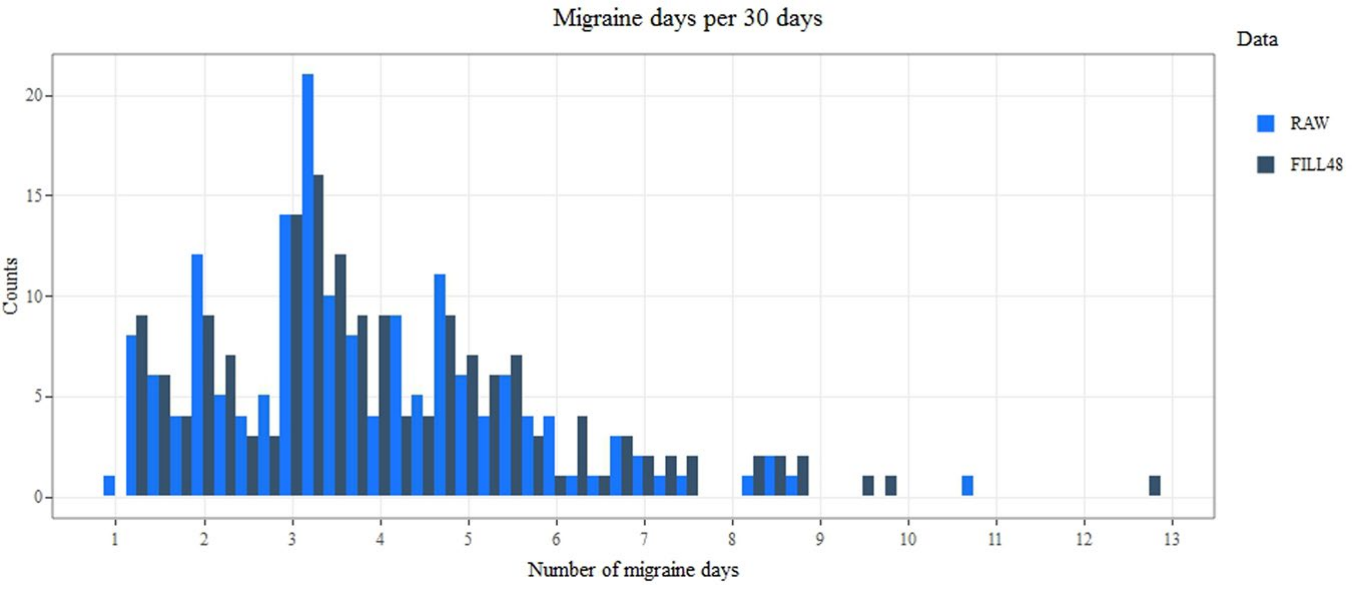
Histogram of the individual frequencies of migraine days pre- (raw) and post (Fill48) imputation of migraine locked days. Migraine frequency is the mean number of migraine days per 30 days.

In Fig. 3 the distribution of individual migraine day frequencies is shown for the raw- and the Fill48-data. The mean frequency of migraine days was estimated to 3.8 days per 30 days on the raw data, and 4.0 on the imputed data.

### Transition probabilities

In the raw diaries, the longest duration migraine attacks observed were three attacks of 9 days each. In the imputed diaries, there was one attack of 10 days duration. This meant that we could estimate TPs *δ*_*i*_ for *i* = 1, …, 9 with the raw data, and for *i* = 1, …, 10 for the imputed data. The overall estimated TPs for the pooled set of diaries are given in Table 4.

**Table 4:** Estimated overall transition probabilities.

TP	Raw Data			Fill48 Data		
	$N$	Estimate	(95% CI)^✠^	${\bf{N}}$	Estimate	(95% CI)^✠^
$\hat{\mu }$	15 407	0.087	(0.083,0.092)	14 221	0.085	(0.080,0.090)
${\hat{\delta }}_{1}$	1 336	0.420	(0.393,0.447)	1 197	0.505	(0.477,0.534)
${\hat{\delta }}_{2}$	559	0.453	(0.411,0.495)	603	0.575	(0.535,0.615)
${\hat{\delta }}_{3}$	252	0.333	(0.276,0.396)	345	0.432	(0.379,0.486)
${\hat{\delta }}_{4}$	84	0.369	(0.268,0.482)	149	0.497	(0.414,0.579)
${\hat{\delta }}_{5}$	31	0.419	(0.251,0.607)	74	0.446	(0.332,0.566)
${\hat{\delta }}_{6}$	13	0.538	(0.261,0.796)	33	0.485	(0.312,0.661)
${\hat{\delta }}_{7}$	7	0.429	(0.118,0.798)	16	0.375	(0.163,0.641)
${\hat{\delta }}_{8}$	3	1.000	(0.310,1.000)	6	0.667	(0.241,0.940)
${\hat{\delta }}_{9}$	3	0.000^†^	(0.000,0.690)	4	0.250	(0.013,0.781)
${\hat{\delta }}_{10}$	0	—^†^	—^†^	1	0.000^†^$\dagger$	(0.000,0.945)
${\hat{\delta }}_{{\rm{Omni}}}\ddagger$	2 288	0.417	(0.397,0.438)	2 427	0.509	(0.489,0.529)

TP = transition probability; *N* = number of underlying observations for the TP; Estimate = point estimate of TP. ^✠^The 95%CI are estimated as Newcombe^15^. ^†^The maximal attack durations observed were 9 days for the raw data and 10 days for the imputed data, hence ${\widehat{\delta }}_{10}$ could not be estimated for the raw data. Furthermore, the last transition probability estimated must be zero (determined by the data). ^‡^This omnibus TP is the probability that an attack continues once begun, without conditioning on the duration.

There was a marked difference in the estimated TPs for *μ* versus the *δ*_*i*_’s. The *μ* was estimated to $\widehat{\mu }=0.087$ on the raw data set, and as 0.085 on the imputed data set, meaning that on a healthy day (in state *S*_*W*_) the risk of migraine attack onset was 8.5% (8.7%) in the imputed (raw) data. Meanwhile, ${\widehat{\delta }}_{1}$ were 0.505 and 0.420 respectively, so that the probability of seeing a second day of migraine, *conditional on having observed one day already*, was significantly elevated compared to the migraine onset probability ($\widehat{\mu }$).

However, Table 4 raises the question of whether the *δ*_*i*_’s depend on *i*? No significant association between TPs and length of attack was found, neither with nor without imputing migraine locked days, with *p* = 0.35 (*p* = 0.59) from an *N*-weighted linear regression ${\widehat{\delta }}_{i} \sim \,i$ on the imputed (raw) numbers.

**Figure 4:**
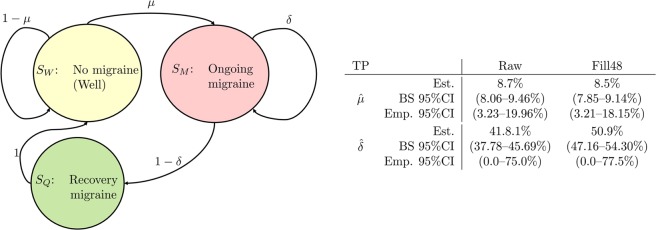
The preferred simple Markov chain model (TP estimates from Table 4) for the progression of migraine attacks, with the estimated overall omnibus transition probabilities. If the Fill48 method is not employed, the state *S*_*Q*_ should be omitted. There is evidence for inter-patient heterogeneity for the *δ*-parameter, while the observed *μ*_*j*_’s appear to be more closely collected around the population mean in this data; see the subsection Individual transition probabilities for the estimation of the bootstrap (BS) 95% CIs.

Taken together, the above suggests that a simple three-state Markov chain (see Fig. 4) might serve as a *good-enough* approximation for modelling the progression of a patient’s migraine attacks. That is: if all the *δ*_*i*_’s are approximately equal, then the Markov chains in Figs. 1 and 4 are equivalent.

### Individual transition probabilities

**Figure 5:**
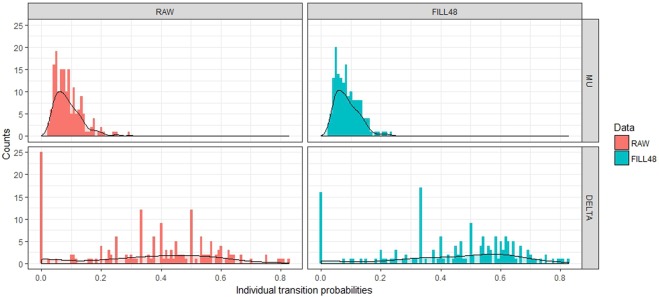
Individual empirical transition probabilities for *μ*_*j*_ and *δ*_Omni,*j*_. Bars represents counts, the curves are kernel density estimate curves^24^. We see a narrow distribution of the *μ*_*j*_’s (top panels) while the point estimates for the *δ*_Omni,*j*_’s (bottom panels) appear more scattered.

We next assessed inter-patient heterogeneity in the TPs, i.e. how well does the simple Markov chain in Fig. 4 approximate an individual patient? After all, the omnibus TPs (Table 4 & Fig. 4) are *overall* TPs—estimated by pooling data from all the diaries. However, the distributions of the *individual* ${\widehat{\delta }}_{{}_{{\rm{Omni}}},j}$’s display non-negligible variance (Fig. 5).

The 165 ${\widehat{\mu }}_{j}$’s display a fairly concentrated distribution: 95% of the individual observations fall within 3–18% (when the Fill48 is applied; 3–20% for the untrimmed diaries.) However, for the 165 ${\widehat{\delta }}_{{\rm{Omni}},j}$’s the corresponding range is 0–78% (0–75%), with median value 50% (40%).

This observation, together with the substantial variation in the individual diaries lengths (no. of observed days for each individual), prompted carrying out a sensitivity analysis to assess the uncertainty of the pooled $\widehat{\mu }$ and $\widehat{\delta }$ parameters. To this end, we performed a *boot strap* (BS) analysis by drawing 10^5^ random samples *with replacement* of size *n* = 165 from the set of 165 diaries, and repeated the procedure for estimating $\widehat{\mu }$ and ${\widehat{\delta }}_{{\rm{Omni}}}$^20^. The resulting 95% BS CIs—the inter 2.5% and 97.5% percentile range of the 10^5^ estimates of the parameters $\widehat{\mu }$ and $\widehat{\delta }$—are given in Fig. 4.

## Discussion

Diaries from patients with episodic migraine demonstrate clustering of migraine days suggesting that the probability of recording a migraine headache on any given day is dependent on the presence of migraine headache on the preceding day:

*P*(*d*_*i*+1_ = migraine∣*d*_*i*_ = migraine) ≠ *P*(*d*_*i*+1_ = migraine∣*d*_*i*_ = migraine free). 

Thus, for research focusing on statistical identification of migraine triggers or on the pathophysiological development of a migraine attack, *it is necessary to account for this clustering of headache days*. Furthermore, our results suggest that a fairly simple Markov chain model with only two active states (Fig. 4) can be used as a mathematical model for the progression of a migraine attack.

Note that under the assumption of independence of attacks (*μ* = 1 − *δ*_*i*_ for all *i*) the model is equivalent to a Markov chain with states *S*_*W*_ and *S*_1_. This means that the Markov chain-approach proposed here—omitting *S*_*Q*_—subsumes the model (implicitly) assumed by Marcus *et al*. and Barra *et al*. as a special case^1,2^.

The Markov chain above might also be overly simplistic but it is much more versatile than assuming a two-state model for attacks. Furthermore, statistics computed for the model presented here provide more robust tests for associations between migraine attack patterns and other triggers than the models previously published. In particular, the Markov chain provides a model to which the probability criterion from Barra et al can be adapted^2^, without increasing Type I errors.

More research will be needed to investigate the TPs, and how they vary across patient subgroups. In particular, our analysis was undertaken on women with episodic migraine whose mean age was 40.8 years, so may not be applicable to attacks in other age groups or to men with episodic migraine. Also, the number of diaries is not very high (*N* = 165), and it would be desirable to validate the model on a larger data set. Obtaining high-quality diary data has traditionally been difficult. We do expect that the advent of smart-phone applications and other digital means for collecting headache data become available, future studies can be conducted to obtain better and more accurate estimates. However, women typically out-number men in clinical trials of migraine, reflecting the higher prevalence of migraine in women and greater disability. Further, migraine attacks are of longer duration in women compared to men^21^, so the management of clustering of days is likely to be more relevant to clinical trials of migraine in women.

Secondly, we found that imputing migraine-locked days appeared to leave the data with high face-validity, and with low impact on other key statistics (mean number of headache days/attack frequency). Our findings support that a single migraine-locked day is part of the same migraine attack but we have only limited pathophysiological data to support this, as presented above. Clinically, patients report that treatment can be effective on headache and associated symptoms but the migraine attack continues to progress. In the case of headache returning after initial effective treatment, this is defined as relapse. The duration of relapse is arbitrarily defined as up to 48 hours after at least 2 hours of pain freedom following medication in the current guidelines^6^. Our results support this definition as freedom symptoms for less than 48 hours spans a single migraine-locked day.

We have taken 48 hours as the quarantine time in order to define discrete episodes but this is based on the duration of premonitory symptoms and limited studies assessing pathophysiological changes before onset to headache. However, we have not accounted for post headache pathophysiology and the duration of attack resolution before the next migraine can be triggered. It is likely that 48 hours is inadequate and 72 hours is a more realistic quarantine between discrete attacks. This will remain an uncertainty until we have more data on attack progression and resolution.

We stress here that the utility of the presented Markov model is its simplicity. What we are seeking is a model that *does not* rely on advanced statistics, or the analysis of other possible covariates that may explain individual variation in the transition probabilities. Our model is intended first and foremost for the analysis of an individual patient’s diary. Examples could be a patient suspecting that consumption of chocolate trigger migraine attacks, or that attack duration is prolonged by barometric pressure. Robust statistical tests—enabling the researcher or clinician to control the level of spurious association—are available as outlined by Marcus et al. and Barra et al. Unfortunately, these authors rely on the assumption of non-clustering of attacks^1,2^. Our conceptual model overcomes this challenge, and such questions can now be framed as whether or not the individual’s transition probabilities are conditional on the presence of the trigger: in the chocolate-trigger example by conditioning *μ*; in the barometric pressure-prolongation scenario by conditioning *δ*.

Furthermore, our model can greatly facilitate estimates of migraine’s impact on disease burden^22,23^. Given that more robust estimates on the transition probabilities (and their inter-patient distributions) can be procured, national estimates for disease burden can be generated with accompanying confidence intervals.

Only patients with *episodic* migraine were included in this study. These are the patients for which the issue of identifying discrete migraine attacks is most pertinent. Our results suggest that the main difference between migraineurs is the propensity of their attacks to *continue*, rather than their propensity for having an attack onset, but this question needs further investigation.

Despite obvious limitations, we recommend the Markov models described here for modelling the attack-progression of migraineurs. In particular, for research on suspected migraine triggers, our model facilitates tests against the null hypothesis that the migraine onset probability *μ* is constant during exposure to the trigger of interest^3^. However, the use of this simple model for modelling episodic disease requires some caution. Validating that the *δ*_*i*_’s an be collapsed onto a *δ*_Omni_ will be important when the *δ*-transitions are the focus. Furthermore, it may be preferable to draw the *δ*’s from a distribution in a simulation study, since our data demonstrated substantial heterogeneity for this parameter.

## Conclusions

We have specified a simple Markov chain-model for the transition of migraine attacks. This model, together with the concept of migraine-locked days, accommodates managing clusters of attacks by imputing migraines onto migraine-locked diary days (the *Fill48* method for pre-treating the diary data).

Migraine days do cluster—equivalently—the probability of migraine on any given day is not independent of migraine on the previous day in our data (*μ* ≠ *δ*). Hence, previously published methods on migraine assuming independence of attacks^1,2^, should be used with caution.

Furthermore, our results suggest that the main source of variation in the patients’ migraine frequencies (the fraction of days with a recorded headache) is driven by variation in the duration of attacks, rather than variation in the migraine onset probabilities. This finding should be considered when conducting research that relies on identifying discrete migraine attacks; particularly when pathophysiology or migraine attack triggers are under scrutiny.

Rudimentary validation yields satisfactory model fit. Hence, the extensive repository of statistical methods appropriate for the analysis of Markov chains is available for diary-based research on patterns of episodic migraine, resulting in a simple, yet theoretically well-founded, method for quantifying the number and duration of migraine attacks. Jointly, this enables researchers and clinicians to procure data of high inter-study validity.

## Data Availability

The datasets generated during and/or analysed during the current study are available upon reasonable request from the Research-Gate© repository https://www.researchgate.net/publication/335577175_Headache_Diaries_DATABASE.
